# Modulating Neuromorphic Behavior of Organic Synaptic Electrolyte-Gated Transistors Through Microstructure Engineering and Potential Applications

**DOI:** 10.1021/acsami.4c05966

**Published:** 2024-07-26

**Authors:** Fu-Chiao Wu, Chun-Yu Chen, Yu-Wu Wang, Chun-Bin You, Li-Yun Wang, Jrjeng Ruan, Wei-Yang Chou, Wei-Chih Lai, Horng-Long Cheng

**Affiliations:** † Department of Photonics, Meta-nanoPhotonics Center, National Cheng Kung University, Tainan 701, Taiwan; ‡ Institute of Photonics, National Changhua University of Education, Changhua 500, Taiwan; § Department of Materials Science and Engineering, National Cheng Kung University, Tainan 701, Taiwan

**Keywords:** organic semiconductors, ion−gels, insulating polymers, polyblends, electric double layers, neuromorphic computing, charge transport, memory effect, logic gates

## Abstract

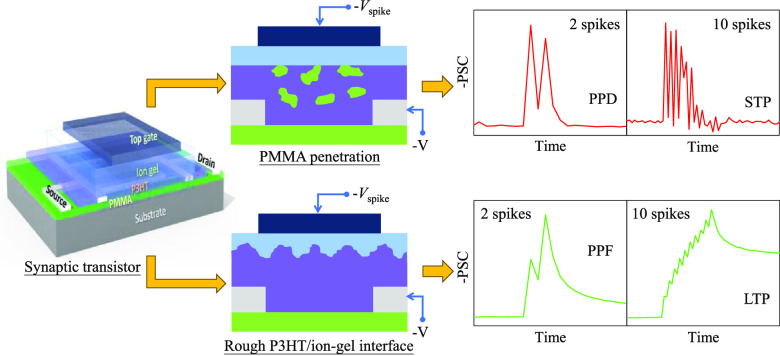

Organic synaptic transistors are a promising technology for advanced electronic devices with simultaneous computing and memory functions and for the application of artificial neural networks. In this study, the neuromorphic electrical characteristics of organic synaptic electrolyte-gated transistors are correlated with the microstructural and interfacial properties of the active layers. This is accomplished by utilizing a semiconducting/insulating polyblend-based pseudobilayer with embedded source and drain electrodes, referred to as PB-ESD architecture. Three variations of poly(3-hexylthiophene) (P3HT)/poly(methyl methacrylate) (PMMA) PB-ESD-based organic synaptic transistors are fabricated, each exhibiting distinct microstructures and electrical characteristics, thus serving excellent samples for exploring the critical factors influencing neuro-electrical properties. Poor microstructures of P3HT within the active layer and a flat active layer/ion–gel interface correspond to typical neuromorphic behaviors such as potentiated excitatory postsynaptic current (EPSC), paired-pulse facilitation (PPF), and short-term potentiation (STP). Conversely, superior microstructures of P3HT and a rough active layer/ion–gel interface correspond to significantly higher channel conductance and enhanced EPSC and PPF characteristics as well as long-term potentiation behavior. Such devices were further applied to the simulation of neural networks, which produced a good recognition accuracy. However, excessive PMMA penetration into the P3HT conducting channel leads to features of a depressed EPSC and paired-pulse depression, which are uncommon in organic synaptic transistors. The inclusion of a second gate electrode enables the as-prepared organic synaptic transistors to function as two-input synaptic logic gates, performing various logical operations and effectively mimicking neural modulation functions. Microstructure and interface engineering is an effective method to modulate the neuromorphic behavior of organic synaptic transistors and advance the development of bionic artificial neural networks.

## Introduction

In recent days, the data processing speed of computers has become faster than ever. Current architectures of computers are based on the von Neumann architecture, which is composed of separate processing and memory units. Data should be transferred between the processing and memory units. However, the data transmission rate between processing and memory units is lower than the data computing speed of the processing unit, thereby limiting the efficiency advancement of computers, the so-called von Neumann bottleneck.^1−3^ Devices simultaneously possessing computing and memory functions have been developed. In biological nervous systems, synapses can complete signal processing and memory simultaneously and then pass this information to the next level of neurons. Devices based on memristor or transistor structures have been adopted to study neuromorphic electrical characteristics and emulate the behavior of biological synapses.^4−8^ Transistors have the advantages of higher signal-to-noise ratio and signal amplification and lower power consumption than memristors. Transistors are three-terminal devices and can read information (such as the postsynaptic potential in a synapse) corresponding to the incoming signal (such as the presynaptic potential in a synapse) concurrently, similar to the working mechanism of biological synapses. Hence, transistors are considered a promising technology for neuromorphic devices. Organic/polymer materials offer several advantages, including low cost, lightweight, flexibility, designable chemical structures, and ease of processing at low temperatures compared with materials like silicon. Furthermore, organic/polymer semiconductors exhibit superior biocompatibility compared to inorganic semiconductors, making them attractive for bioelectronic device applications. Consequently, numerous studies have explored the use of organic/polymer semiconductors in synaptic transistors that mimic biological synapses,^9−14^ enabling new possibilities in neuromorphic computing, artificial intelligence, and brain–machine interfaces.

Various methods have been adopted to fabricate organic synaptic transistors to achieve concurrent computing and memory functions. Ion–gels and electrolytes are used as dielectric layers to form electric double layers (EDLs) and/or cause ion doping in active layers to generate memory effect of synaptic devices.^15−19^ Ferroelectric materials are selected as dielectric layers to lead memory function of synaptic devices through the dipolar polarization effect.^20−22^ Charge capturing or electret layers are adopted as dielectrics to store charge carriers and result in memory behavior of synaptic devices.^23−27^ Functional heterostructures are designed for layered dielectric or active layers to trap charge carriers and induce the memory effect of synaptic devices.^28−30^ Functional organic semiconductor materials are synthesized to change the degree of ion doping in active layers and manipulate memory behavior of synaptic devices.^31,32^ However, most works focus on features of dielectric layers correlated with neuromorphic electrical characteristics of organic synaptic transistors and their application in artificial neural networks.^10,18,21−26,28^ The significant impact of microstructural and interfacial properties on the electrical characteristics of organic thin-film transistors (TFTs) has been extensively studied and demonstrated, with an expected similar influence on the neuromorphic electrical properties of organic synaptic transistors. Therefore, a comprehensive understanding of how microstructures of active layers and the interface between the active layer and the dielectric layer modulate the neuromorphic behavior of organic synaptic transistors is needed to optimize device performance and emulate the diverse behavior of biological synapses.

In various neuromorphic behavior of organic synaptic transistors, excitatory postsynaptic current (EPSC) and inhibitory postsynaptic current (IPSC) are two commonly observed behavior under the stimulation of single spike.^2,12,14,21,22,26^ Under a paired-spike, paired-pulse facilitation (PPF) is a usual behavior of organic synaptic transistors.^2,7,14,19−22,30−32^ Stimulated by multiple spikes, organic synaptic transistors often can perform short-term potentiation (STP), short-term depression, long-term potentiation (LTP), and long-term depression behavior.^2,15,17,19,20,27^ Opposite behavior such as excitatory and inhibitory as well as potentiation and depression can be achieved by providing devices spikes with opposite polarities or different intensities.^12,17,19−22,26,28^ A neuromorphic behavior, paired-pulse depression (PPD), is seldom observed in organic synaptic transistors.^19,28^ In biological organisms, the PPD behavior of synapses generally exists in the nervous system and plays an important role in many biological mechanisms, including perceptual adoption process, sound localization, enhancement of information transmission efficiency, and regulation of energy use.^33−35^ A specific range of spike voltage is selected to stimulate organic synaptic transistors with a multilayered dielectric and confer them with PPD behavior.^28^ However, in face of the same incoming signal, concurrent PPF and PPD behavior of synapses is often required in biological mechanisms, such as the auditory nervous system for sound localization.^36,37^ Organic synaptic transistors that perform PPD behavior should be developed to comprehensively simulate biological neuromorphic behavior and advance bionic artificial neural networks.

In this study, we fabricated organic synaptic electrolyte-gated transistors for neuromorphic applications, employing a semiconducting/insulating polyblend-based pseudobilayer with embedded source and drain electrodes as the active layer, referred to as the PB-ESD architecture. The semiconductor material chosen was poly(3-hexylthiophene) (P3HT), while the insulator was poly(methyl methacrylate) (PMMA). Previous research has shown that the P3HT/PMMA PB-ESD architecture offers advantages in achieving superior electrical performance and quasi-stable continuous long-term operation characteristics compared to only P3HT-based transistors with SiO_2_ dielectrics.^38^ In this work, three different process conditions for P3HT were employed to create the corresponding PB-ESD active layers for organic synaptic transistors, resulting in distinct neuromorphic electrical characteristics. We investigated the correlations between charge behavior and the microstructural and interfacial features of various P3HT/PMMA PB-ESD-based devices and discussed the corresponding mechanisms of neuromorphic behavior in different synaptic transistors. Additionally, with the inclusion of a second bottom gate electrode, these P3HT/PMMA PB-ESD-based devices can function as two-input synaptic logic gates capable of performing diverse logical operations based on their synaptic behavior.

## Experimental Section

### Device Fabrication

The fabrication of P3HT/PMMA PB-ESD-based synaptic transistors was started on a clean silicon wafer as a substrate. PMMA (average molecular weight: 996 000, Sigma–Aldrich) dissolved in *p*-xylene at a concentration of 2 wt % was spin coated on the silicon wafer and baked at 120 °C for 2 h to serve as a modification layer. 80 nm silver as source and drain electrodes was thermally evaporated on PMMA via a patterned shadow mask to define a channel length of 200 μm and a channel width of 2000 μm. For the fabrication of the active layers with different microstructures, three kinds of P3HT processes were adopted.

**Figure 1:**
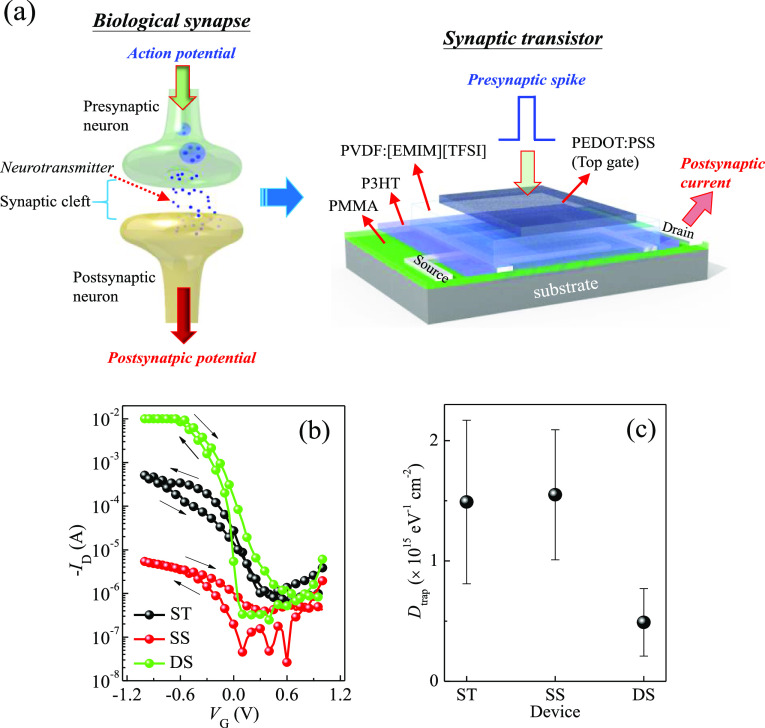
(a) Illustration of the P3HT/PMMA polyblend-embedded source drain (PB-ESD) architecture for synaptic transistors, structurally mimicking typical biological synapses. (b) Transfer curves (*V*_D_ = −0.5 V) and (c) *D*_trap_ for various P3HT/PMMA-based transistors.

First, P3HT (average molecular weight: 58 000, RMI-001E, Rieke Metals) dissolved in *p*-xylene at a concentration of 0.34 wt % was spin coated onto the PMMA and annealed at 120 °C for 2 h, denoted as the ST device (spin coating and thermal annealing processes). Second, 0.1 wt % P3HT in *p*-xylene was spin-coated onto the PMMA and treated with a solvent annealing process for 12 h, referred to as the SS device (spin coating and solvent annealing processes). Third, 0.1 mL of P3HT solution, 0.1 wt % in *p*-xylene, was drop-casted onto the PMMA and treated with a solvent annealing process for 12 h, denoted as the DS device (drop casting and solvent annealing processes). Next, polyvinylidene difluoride (PVDF, average molecular weight: 275 000, Sigma-Aldrich) blended with 1-ethyl-3-methylimidazolium bis(trifluoromethylsulfonyl)imide ([EMIM][TFSI], Iolitec GmbH) in acetone was spin coated onto a warm glass substrate and baked at 140 °C for 12 h to form a PVDF:[EMIM][TFSI] ion–gel film. A piece of the ion–gel film was cut and peeled off, then stuck onto the P3HT to serve as a dielectric layer. Finally, 1 mL of poly(3,4-ethylenedioxythiophene):poly(styrenesulfonate) (PEDOT:PSS) solution (PH1000, CleviosTM) was drop-casted onto a glass substrate and baked at 80 °C for 12 h. A piece of the PEDOT:PSS film was cut and peeled off in ethylene glycol, then rinsed with ethanol. After drying, the peeled PEDOT:PSS film as a gate electrode was stuck onto the ion–gel layer to complete a synaptic transistor device, as shown in Figure 1a.

### Characterization

Electrical measurements of organic synaptic transistors were implemented in a nitrogen-filled glovebox and using a Keithley 4200 semiconductor characterization system. Absorption spectra were acquired by a GBC Cintra 202 UV–Vis spectrometer with a spectral resolution of 0.9 nm. X-ray diffraction (XRD) spectra were obtained through a Bruker D8 Discover X-ray diffractometer with an X-ray wavelength of 0.154056 nm. For the calculations of surface energy of solid specimens, water and diiodomethane were adopted as polar and nonpolar liquids, respectively, and their contact angles on solid specimens were measured via a DataPhysics OCA 15plus optical contact angle measuring and contour analysis system. Surface morphologies were investigated using a Park XE-100 atomic force microscope (AFM) system with a PPP-NCHR (Nanosensors) probe model and a scan rate of 0.5 Hz.

## Results and Discussion

### Electrical Characterization of TFTs

Figure 1b shows the typical transfer curves of various P3HT/PMMA PB-ESD-based transistor devices. For the SS and DS devices, their output drain current (*I*_D_) during the forward gate voltage (*V*_G_) sweep, going from positive to negative voltage, is lower compared to the reverse *V*_G_ sweep. During forward *V*_G_ sweep, the oppositely charged ions in the ion–gel dielectric are polarized and form an electric double layer (EDL) at the interface of P3HT with ion–gel dielectric.^1,3,6,17,19^ In addition, the dipoles of PMMA and ferroelectric PVDF in ion–gel are polarized.^21,22,39,40^ These phenomena can enhance hole accumulation in the active channel of P3HT. During the reverse *V*_G_ sweep, the elimination of the EDL and polarized dipoles is slow, and this residual effect causes the *I*_D_ of the SS and DS devices to be higher compared to the forward *V*_G_ sweep. Conversely, the ST device exhibits a lower *I*_D_ during the reverse *V*_G_ sweep than during the forward *V*_G_ sweep. For the ST device, during the forward *V*_G_ sweep, the gate-bias stress can induce the formation of trap states in the active P3HT channel, hindering charge transport and leading to a decreased *I*_D_ value in the subsequent reverse *V*_G_ sweep. Hence, the gate-bias stress effect in the ST device is dominant compared to the SS and DS devices.

Among these devices, the DS device exhibits the highest *I*_D_, reaching the compliance limit of 10 mA at a *V*_G_ of only −0.7 V, as well as the sharpest *S* characteristics with a low value of 0.095 ± 0.021 V/dec calculated from the forward *V*_G_ sweep. A comparative table of the electrical parameters of these devices can be found in Table S1. The SS device shows the lowest *I*_D_ of 10^–4^ −10^–5^ A, while the ST (*S* = 0.168 ± 0.049 V/dec.) and the SS (*S* = 0.173 ± 0.039 V/dec.) devices have comparable *S* values. The trap density (*D*_trap_) of active channels in devices can be estimated using the following equation:^21,23,41,42^
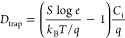
1where *k*_B_ is the Boltzmann constant, *T* is the temperature, *q* is the elementary charge, and *C*_i_ is the capacitance of the dielectric layer. As shown in Figure 1c, the *D*_trap_ of the DS device is lower than those of the two other two devices, leading to efficient charge transport and a higher *I*_D_ for the DS device. This high *I*_D_ at *V*_G_ greater than −0.6 V corresponds to an extremely high channel conductance (*G*) value of nearly 100 S/m, which is 25 times and 2,500 times higher than the ST and SS devices, respectively (Figure S1). The ultrahigh *G* value could be attributed to an increase in charge carrier density in the channel due to the high capacitance of the electrolyte gate dielectric.^43^ The *V*_t_ values of the ST and SS devices remain nearly unchanged under forward and reverse *V*_G_ sweeps (Figure 1b). However, for the DS device, the *V*_t_ under the reverse *V*_G_ sweep exhibits a positive shift (0.12 ± 0.06 V) compared to that of the forward *V*_G_ sweep, signifying the occurrence of a memory effect.

### Microstructural Characterization of Active Layers

**Figure 2:**
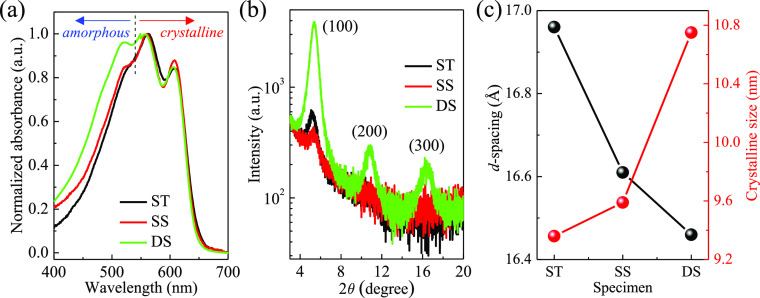
(a) Absorption spectra, (b) XRD spectra, and (c) *d*-spacing and crystalline sizes of different P3HT specimens.

The microstructural characteristics of the semiconducting P3HT within the various PB-ESD architectures were investigated by using UV–Vis absorption spectroscopy and X-ray diffraction. Figure 2a shows the normalized absorption spectra of various specimens. The absorption region of P3HT above 540 nm is considered the absorption mainly from crystalline P3HT and below 540 nm reflects the absorption mostly from amorphous P3HT.^39,44^ The differences in the shape of the absorption peaks of crystalline P3HT among the three types of specimens are small. However, regarding the ratio of amorphous to crystalline absorbance of the P3HT, the DS specimen shows a higher value compared with the ST and SS specimens, suggesting a larger amorphous fraction. However, it should be noted that the DS specimen was prepared using the drop-casting method followed by solvent annealing, resulting in a significantly thicker film compared to those of the ST and SS samples. Consequently, the initial crystalline absorption intensity of the DS specimen was much higher than that of the other two samples, by approximately 10 times (Figure S2).

For further investigation on the crystalline P3HT portion, the XRD spectra of different P3HT specimens were recorded. As shown in Figure 2b, all kinds of specimens possess a diffraction peak at 2θ of around 5.3°, reflecting the (100) lattice plane of lamellar supramolecular structures from edge-on P3HT molecules stacking along the *a*-axis (out-of-plane direction).^39,44^ Compared with the other two kinds of specimens, two additional high-order diffraction peaks are observed in the DS specimen, signifying that the DS specimen has a better quality of crystalline structures. Based on the position of (100) diffraction peak, the *d*-spacing value of stacked P3HT molecules can be calculated by Bragg’s law. As plotted in Figure 2c, the DS and ST specimens have shorter and longer *d*-spacing values, respectively, indicating that the stacking of P3HT molecules is more compact in the DS specimen and is looser in the ST specimen. The crystalline size (*L*_cry_) of different P3HT specimens can be estimated using the Scherrer equation:^44,45^
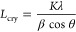
2where *K* is the Scherrer constant and a value of 0.9 is used,^44,45^ λ is the wavelength of X-ray, β is the half-width of a diffraction peak, and θ is the position of a diffraction peak. As shown in Figure 2c, the DS specimen has a larger *L*_cry_ than the other two kinds of specimens. In the crystalline P3HT portion, the DS specimen possesses better crystalline quality, larger *L*_cry_, and denser molecular stacking, resulting from longer self-assembling time of P3HT molecules via joint drop-casting and solvent annealing processes,^46,47^ compared with the ST and SS specimens. These features are beneficial for charge transport. Therefore, a higher *I*_D_ is observed in the DS device. In addition, poor microstructures of crystalline P3HT (small *L*_cry_ and loose molecular stacking) are observed in the ST specimen, causing the ST device to be easily influenced by gate-bias stress, resulting in lower *I*_D_ in the reverse *V*_G_ sweep than in the forward *V*_G_ sweep (Figure 1b).

**Figure 3:**
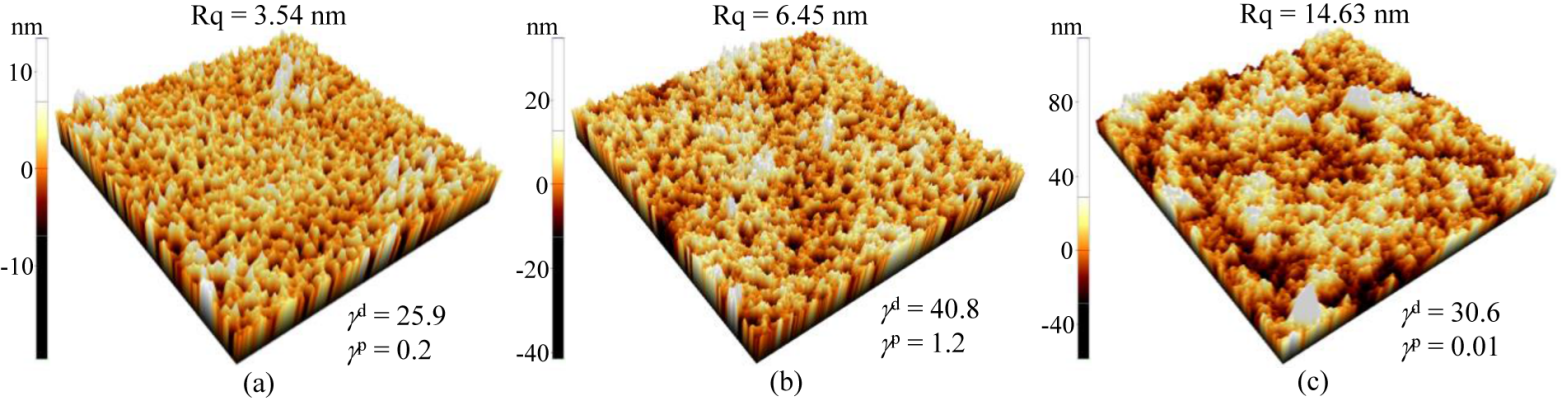
AFM images (20 μm × 20 μm) of (a) ST, (b) SS, and (c) DS specimens. The root–mean–square surface roughness (Rq) and dispersive and polar surface energy components (γ^*d*^ and γ^*p*^, respectively, in unit of mJ/m^2^) are also shown.

Although the SS device has larger *L*_cry_, denser molecular stacking in the crystalline structure, and comparable *D*_trap_, its *I*_D_ value is lower than that of the ST device (Figure 1b). Considering that the active channels of devices are located near the surface of the top P3HT layers (Figure 1a), we further investigated the surface features of different P3HT/PMMA PB-ESD specimens. Surface energy of a material is composed of dispersive and polar surface energies (γ^*d*^ and γ^*p*^). The values of γ^*d*^ and γ^*p*^ can be calculated through the Owens–Wendt geometric mean equation:^29,42^


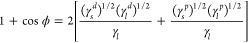
3where ϕ is the contact angle of a liquid on a solid, γ is the surface energy equal to γ^*d*^ + γ^*p*^, and suffixes *s* and *l* denote solid and liquid, respectively. Droplets of polar and nonpolar liquids with known γ^*d*^ and γ^*p*^ values on a solid specimen were measured to acquire ϕ values. With ϕ, γ^*d*^, and γ^*p*^ values of the two liquids, the γ^*d*^ and γ^*p*^ values of a solid specimen were obtained through eq 3. The γ^*d*^ and γ^*p*^ values are shown in Figure 3. The γ^*d*^ and γ^*p*^ values of the ST specimen are close to those of the DS specimen. Both ST and DS specimens have different γ^*d*^ and γ^*p*^ values from the PMMA specimen (γ^*d*^ = 36.2 mJ/m^2^; γ^*p*^ = 7.7 mJ/m^2^). This result reflects that the main component at the surface of the ST and DS specimens is P3HT. Interestingly, the SS specimen shows γ^*d*^ and γ^*p*^ close to the PMMA and the ST specimens, respectively. This result signifies that the surface of the SS specimen comprises PMMA and P3HT components. For the SS specimen, during solvent annealing of the top P3HT layer, the underlying PMMA could permeate into the P3HT layer and reach near the surface, resulting in the coexistence of PMMA and P3HT at the surface. Although solvent annealing was also used in the DS specimen and the penetration of PMMA could occur, the thick P3HT top layer from the drop-casting process made PMMA hard to reach near the surface. Hence, in the SS device, the insulating PMMA located near the P3HT active channel can hinder charge transport significantly, leading to a much lower *I*_D_ value in comparison with the ST device. Figure 3 shows AFM images of various P3HT/PMMA PB-ESD specimens. The XRD analysis indicates that the crystalline structure of P3HT adopts an edge-on conformation. Therefore, the elevated regions observed in the AFM images can be attributed to the growth of crystalline structures, while the lower regions correspond to areas with a higher proportion of amorphous content. Compared to the other two specimen types, the DS specimen exhibits a significantly higher surface roughness (Rq) due to the presence of much larger crystalline domains in the P3HT layer. The high Rq of the DS specimen slows the formation of the hole channel in the DS device, leading to a more negative *V*_t_, as evident from the transfer curve at forward *V*_G_ sweep shown in Figure 1b. Moreover, the high Rq leads to a large interfacial area of crystalline P3HT with ion–gel dielectric, causing more ions to accumulate at the interface.^48,49^ The high Rq also can induce a large local electric field to promote the separation of oppositely charged ions.^50^ These phenomena contribute to the formation of a strong EDL with high capacitance at the P3HT/dielectric interface, leading to a very high *I*_D_ even under extremely low operating voltages (only −0.5 V) in the DS device. The Rq of the SS specimen is slightly higher than that of the ST specimen. Besides the slightly larger *L*_cry_, the penetration of PMMA can increase the Rq of the SS specimen due to phase separation.^39^ As a result, the formation of a hole channel in the ST device is easier than in the SS device. During forward *V*_G_ sweeps, a more positive *V*_t_ is typically observed for the ST device, compared to the SS device, as shown by the representative data in Figure 1b and Table S1.

### Artificial Synaptic Characteristics of TFTs

**Figure 4:**
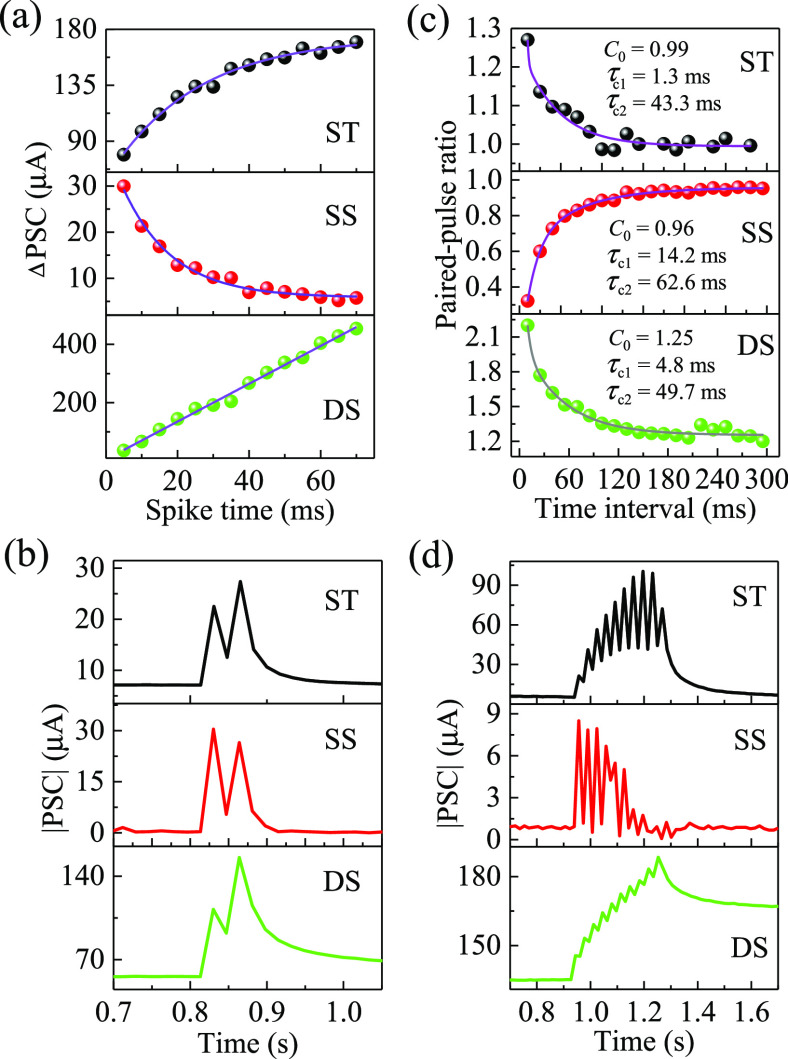
Electrical characteristics (with both drain and spike voltages set to −0.5 V) of various P3HT/PMMA PB-ESD synaptic transistors. (a) Postsynaptic current (PSC) variations of devices under various spike durations. (b) PSC variations of devices stimulated by a paired-spike. The fitting parameters *C*_0_, τ_c1_, and τ_c2_ obtained from eq 4 are also shown. (c) Paired-pulse ratio (PPR) variations of devices under the stimulation of a paired-spike (spike duration of 5 ms) with various time intervals. (d) PSC variations of devices stimulated by 10 spikes. Note: Solid lines in parts (a) and (c) represent fitting curves. For the paired-spike stimulation in (b) and the multiple-spike stimulation in (d), the spike duration and time interval between spikes were 5 and 25 ms, respectively.

In TFTs, the giving of *V*_G_ pulse can cause potential change in the active channel and result in the variation of the *I*_D_ value. This mechanism is like biological synapses where the action potential in the presynaptic neuron triggers a potential change in the postsynaptic neuron, as illustrated in Figure 1a. Therefore, TFTs can emulate behaviors of biological synapses. The synaptic electrical characteristics of various P3HT/PMMA PB-ESD TFTs were investigated. Figure 4a compares the postsynaptic current (PSC, namely, *I*_D_) variations as a function of various spike (*V*_G_) durations under single-spike stimulation for these different organic synaptic transistors. The PSC values of three kinds of devices are increased under the stimulation of a spike, indicating an EPSC behavior (Figure S3). For the ST and the DS devices, the PSC values increase with increasing spike duration (*t*_on_), performing a potentiated EPSC behavior commonly observed in organic synaptic transistors.^10,17,20−22,27,29−31^ The PSC increment of the DS device is greater than that of the ST device, stemming from the more efficient charge transport and stronger EDL formation in the DS device. Furthermore, the DS device also exhibits significant STP even under a very short single-spike stimulation duration (*t*_on_ of 5 ms, Figures S4). However, interestingly, reduced increment of PSC values with increasing *t*_on_ are observed in the SS device (Figure S3b). The SS device exhibits a depressed EPSC behavior. All of these devices demonstrate different spike-time-dependent plasticity behaviors. The ST and DS devices, respectively, follow sublinear and quasilinear growth trends as the spike duration increases, while the SS device exhibits a near-exponential decay behavior. The three distinctly different behavioral trends obtained in Figure 4 can be reproduced in devices from different batches, but there are also inevitable device-to-device (DtD) variations in the EPSC under different *t*_on_ (Figure S3). In terms of the electrical characteristics of the TFTs (Table S1), the SS devices exhibit the worst electrical characteristics and relatively large DtD variations. When given different *t*_on_ (5–70 ms), the standard deviation (σ) of EPSC for SS devices decreases with increasing *t*_on_, reaching a level of approximately 1–10 μA. Although its σ is the smallest among these devices, it indeed has the largest DtD variations in EPSC (reaching an average of 41%), which can be attributed to the PMMA infiltration into the P3HT active layer. On the other hand, the ST and DS devices have similar DtD variations (an average of approximately 20%). When the *t*_on_ is short, the EPSC of the devices is small, and its σ is also smaller (e.g., σ < 10 μA at *t*_on_ = 5 ms). As *t*_on_ increases, producing larger EPSC, it is also accompanied by larger σ between devices.

Figure 4b shows the EPSC variations of different devices stimulated by a paired-spike. The ST and DS devices produce a higher PSC value under the second spike compared to the first spike, reflecting PPF behavior. In contrast, the PSC value of the SS device under the second spike is lower than that under the first spike, indicating a PPD behavior. After stimulation of the second spike, the PSC values of the ST and SS devices decrease rapidly back to the initial state (before stimulation), showing STP behavior. However, the PSC value of the DS device decreases slowly and is maintained at a value above the initial state for a period, meaning LTP behavior. The value of PSC increment under the second spike divided by that under the first spike is defined as the paired-pulse ratio (PPR). Figure 4c plots PPR variations of different devices stimulated by a paired-spike with various time interval. The PPR values of the ST and DS devices are above 1 and decrease with increasing time intervals, a typical phenomenon observed in the PPF behavior of biological synapses. On the contrary, the PPR value of the SS device is below 1 and increases with increasing time interval, a typical phenomenon presented in the PPD behavior of biological synapses. For further investigation of the PPR variation, a biexponential function in the following was adopted to fit the PPR variation curves:^20−22,31,32^
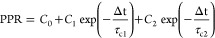
4where *C*_0_ is a constant, *C*_1_ and *C*_2_ are the facilitation (positive) or depression (negative) magnitudes of different phases, Δ*t* is the time interval, and τ_c1_ and τ_c2_ are the characteristic relaxation time of different phases. The fitting parameter values are given in Figure 4c. All kinds of devices have longer τ_c2_ than τ_c1_, signifying that their electrical behavior under a paired-spike stimulation includes rapid and slow phases, similar to that observed in biological synapses.^21,32,51^ The *C*_0_ values of ST and SS devices are close to 1, indicating that their PPR values converge on 1 with an increasing time interval, reflecting an STP behavior. For the DS device, a *C*_0_ of above 1 is obtained, representing that as the time interval increases the PPR converges on a value above 1, reflecting an LTP behavior. In addition, the *C*_1_ and *C*_2_ values of the ST and DS devices are positive, which is a feature of the PPF behavior. However, negative *C*_1_ and *C*_2_ values are obtained in the SS device, which is a feature of the PPD behavior.

**Figure 5:**
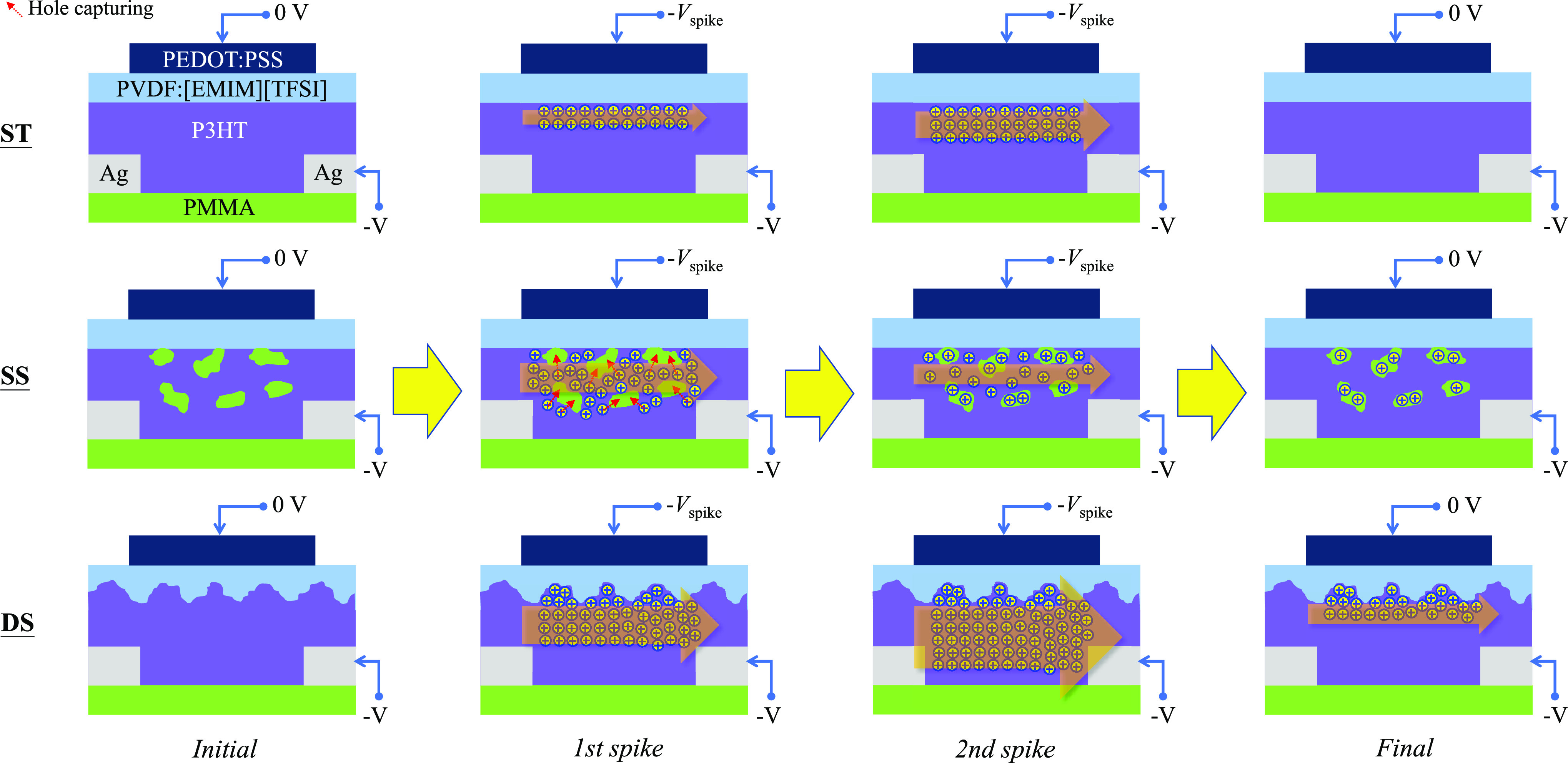
Illustration of working mechanisms of three kinds of the as-prepared organic synaptic transistors during the stimulation of a paired-spike.

The PSC decay curve after the second stimulation (Figure 4b) can be fitted by a triexponential function in the following to analyze the charge release mechanism:^31,32,52,53^
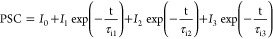
5where *I*_0_ is the PSC before stimulation, *I*_1_, *I*_2_, and *I*_3_ are the magnitudes of PSC from different sources, *t* is the time, and τ_i1_, τ_i2_, and τ_i3_ are the characteristic relaxation time of various PSC sources. The results of fitting parameters are given in Table 1. All those characteristic relaxation time are at above the ms level, which could be relevant to slow elimination of EDL due to slow ion migration after stimulation.^31,32,52^ All kinds of devices have fast relaxation time (τ_i1_) and their τ_i1_ proportion among other relaxation times is the highest, mainly resulting from hole release in the conducting channel. However, the τ_i1_ of the SS device is shorter than those of the other two kinds of devices. Apart from the SS device, both ST and DS devices have a slow relaxation time (τ_i3_). Nonetheless, compared with the DS device, the τ_i3_ of the ST device is shorter and the proportion is lower, which might stem from hole release from the denser trap states in the ST device (Figure 1c). Only the DS device possesses a medium relaxation time (τ_i2_). Compared with the other kinds of devices, the DS device has larger amorphous P3HT portion. The hole mobility in amorphous P3HT regions is lower than crystalline P3HT regions, leading to a slower hole release from amorphous P3HT than from crystalline P3HT. Hence, a hole release time slower than τ_i1_, namely, τ_i2_, can be observed in the DS device. The working mechanisms of various P3HT/PMMA PB-ESD synaptic transistors stimulated by a paired-spike are illustrated in Figure 5. Based on the discussion above, compared with the ST device, the main microstructural feature of the SS device is the distribution of insulating PMMA nearby the P3HT/ion–gel dielectric interface, and those of the DS device are rougher interface of P3HT with ion–gel dielectric and better quality of crystalline P3HT. During stimulation of the first spike, holes accumulate near the P3HT/ion–gel dielectric interface to form a conducting channel, resulting in the occurrence of EPSC of the three kinds of devices. In the SS device, some holes could be captured by PMMA molecules nearby the conducting channel during the first spike.^13,23,54,55^ During the stimulation of the second spike, in the ST and the DS devices, more holes further accumulate in the conducting channel, causing a higher PSC than that under the first spike (PPF behavior, Figure 4b). Furthermore, a larger PSC increment under the second spike, namely, a higher PPR (Figure 4c), can be observed in the DS device compared with the ST device because a rougher P3HT/ion–gel dielectric interface can generate a strong EDL to enhance hole accumulation and better crystalline quality can lead to efficient hole transport. As for the SS device, the captured holes in PMMA can impede hole accumulation in the conducting channel, posing a PSC lower than that under the first spike (PPD behavior, Figure 4b). In addition, as spike time increases, the number of captured holes increases, leading to suppressed hole accumulation and depressed EPSC, as shown in Figure 4a. After the second spike, hole release from the conducting channel makes PSC values of the ST and SS devices decrease to the initial state (STP behavior, Figure 4b). The holes captured by PMMA in the SS device can facilitate hole release from the conducting channel, leading to a fast decrease of PSC. Therefore, a shorter τ_i1_ is observed in the SS device than the ST device (Table 1). For the DS device, the rough interface of P3HT with ion–gel dielectric can help some negative ions remain at the interface after the second spike,^48,49^ resulting in the existence of EDL and residual hole accumulation. Moreover, the large amorphous P3HT portion of the DS device is conducive to the infiltration of negative ions during the stimulation. After the second spike, the migration of negative ions from P3HT back to ion–gel is slow, and the remaining negative ions can keep hole accumulation in the conducting channel. Consequently, the hole release in the DS device is slower than the other two kinds of devices, and a much longer τ_i3_ is observed (Table 1). The remaining hole accumulation keeps the PSC of the DS device higher than the initial state for a period after the second spike (LTP behavior, Figure 4b).

**Table 1:** Fitting Parameter Values for PSC Curves of Various P3HT/PMMA PB-ESD Synaptic Transistors, Obtained from eq 5^a^

	relaxation time (ms)						proportion (%)					
	2 spikes			10 spikes			2 spikes			10 spikes		
device	τ_i1_	τ_i2_	τ_i3_	τ_i1_	τ_i2_	τ_i3_	τ_i1_	τ_i2_	τ_i3_	τ_i1_	τ_i2_	τ_i3_
ST	18.2	n/a	218.2	15.2	75.2	370.9	93.7	n/a	6.3	37.1	54	8.9
SS	11.6	n/a	n/a	n/a	n/a	n/a	100	n/a	n/a	n/a	n/a	n/a
DS	19.8	91.8	826.6	n/a	48.1	13352.9	64.6	24.9	10.5	n/a	12.6	87.4

a τ_i1_, τ_i2_, and τ_i3_ represent the characteristic relaxation time constants of the different PSC sources.

Figure 4d shows PSC variations of the as-prepared synaptic transistors under stimulation with 10 spikes. Like the results in the stimulation of a paired-spike, the PSC value of the SS device decreases with increasing spike numbers and decreases to the initial state after the eighth spike, performing a depressed EPSC behavior. Specifically, no obvious increase in PSC of the SS device is observed during the last two spikes. After the stimulation of 8 spikes, the PMMA regions nearby the conducting channel of the SS device could capture enough holes to completely prevent hole accumulation during the last two spikes, leading to nearly unchanged PSC response. Furthermore, even when the time interval between the spikes (*t*_off_) was increased to 40 ms, the above phenomenon of the PSC value returning to the background level could still be observed (Figure S5a). However, after such prolonged and repeated stimulation, we noted that the PSC response gradually became weaker. This is likely due to the increased number of holes captured by PMMA in the SS device. On the contrary, the PSC values of the ST and the DS devices increase with increasing spike numbers, performing a potentiated EPSC behavior. After the stimulation of 10 spikes, the PSC of the ST device decreases back to the initial state within a short period, similar to the behavior observed after 2 spikes (Figure 4b). This demonstrates a short-term memory process. Nonetheless, the DS device performs a PSC decay far slower than that of the ST device. The remaining PSC of the DS device in the period after 10 spikes is higher than the initial PSC, compared with that after 2 spikes (Figure 4b), demonstrating a long-term memory (LTM) process. The PSC decay curves of the ST and the DS devices after the tenth stimulation were fitted using eq 5 to investigate the hole release mechanisms. Table 1 lists the results of the fitting parameters. The ST device has an additional relaxation time, τ_i2_, compared with that after 2 spikes. The τ_i2_ value is comparable to that of the DS device after 2 spikes (Table 1), signifying that with increasing spike numbers holes become easy to locate at amorphous P3HT portions of the ST device. Moreover, the τ_i2_ proportion becomes the highest among other relaxation times, signifying that the hole release main path is from amorphous P3HT portions. The τ_i1_ and τ_i3_ values of the ST device are comparable to those after 2 spikes (Table 1), indicating the existence of the same hole release paths (from conducting channel and trap states) as those after 2 spikes. These hole release paths are short-term processes, leading to rapid PSC decay of the ST device after 10 spikes. For the DS device after 10 spikes, the τ_i3_ is around 16 times longer than that after 2 spikes and becomes the leading proportion among other relaxation time. After the stimulation of 10 spikes, the rough P3HT/ion–gel dielectric interface facilitates the adhesion of more negative ions and significantly slows hole release time in the conducting channel, compared with that after 2 spikes (Table 1).^48,49^ On the other hand, the large amorphous P3HT portion might allow the infiltration of more negative ions to allow more holes to remain in the conducting channel and reduce the hole release time, in comparison with that after 2 spikes.^19,31^ The leading proportion of τ_i3_ signifies that the main hole release path is from the elimination of negative ions at rough interface and/or amorphous P3HT, which is a long-term process, posing the LTM behavior of the DS device after 10 spikes. Even when the *t*_off_ value was significantly increased (e.g., to 180 ms), the DS device still retained remarkably robust LTP behavior (Figure S5b). In contrast, when *t*_off_ was increased to 40 ms, the ST device exhibited more disordered EPSC characteristics as the number of stimulations increased (Figure S5c). In addition, because most holes in the conducting channel are greatly influenced by the large number of remaining negative ions, no applicable τ_i1_ value is obtained in the DS device. Finally, we also need to mention that for the DS and ST devices, even with the increasing number of operation cycles, their ΔPSC response does exhibit a decreasing trend. However, their synaptic behaviors are still maintained. Moreover, their background current values have gradually increased, reflecting their learning and memory effects, i.e., LTM. This should be reasonable, as it is similar to the process of learning and forgetting in neurons—they cannot completely return to their initial state.

Despite the low concentration of only 0.1 wt % P3HT solution, the DS device not only exhibits excellent electrical performance as a TFT but also possesses good synaptic characteristics. Table S2 presents a comparative study highlighting the superior electrical parameters of the DS device among the reported P3HT-based electrolyte-gated transistors, particularly the near-zero Vth and the very sharp S value, even though this study uses the lowest concentration of the P3HT solution. The subthreshold characteristics of a transistor are a crucial indicator of the gate’s ability to modulate the channel conductance. Table S3 compares various polymer semiconductor-based synaptic transistors, demonstrating that the DS device exhibits excellent PPF characteristics, even under short (5 ms) and small stimulation voltages. In addition to LTP, the DS device also exhibits LTM characteristics. Even after 800 consecutive multiple stimulations, the channel conductance of the device continues to increase steadily without reaching saturation, indicating a large number of memory states and excellent memory capacity (Figure S6). The low concentration of P3HT required, along with the simple fabrication process and inexpensive materials, makes this approach highly advantageous compared to other related works in the field.

### Simulation of Neural Networks

The as-prepared P3HT/PMMA PB-ESD synaptic transistors can also be applied to the simulation of neural networks. Based on the tunable *G* values in the synaptic transistors, a three-layer multilayer perceptron (MLP) artificial neural network (ANN) has been simulated for supervised learning tasks using the Modified National Institute of Standards and Technology (MNIST) handwriting image database (Figure S7a). The ANN consists of 256 input neurons (corresponding to 16 × 16 MNIST data), 100 hidden neurons, and 10 output neurons (corresponding to the 10 digits from 0 to 9). During each training epoch, the ANN was trained on 8,000 randomly selected patterns from a set of 60 000 training images, and the recognition accuracy was tested on a separate set of 10 000 test images. The results show that under −0.5 V stimulation with a time duration of 5 ms for each spike, when the *t*_off_ is short (i.e., 10 ms), the ST device exhibits a high recognition accuracy of nearly 90% (Figure S7b). However, as *t*_off_ increases to 25 ms, the recognition accuracy of the ST device drops significantly, to only 59%. In contrast, the DS device maintains a higher accuracy of 74% even at *t*_off_ = 25 ms and achieves 84% accuracy at *t*_off_ = 10 ms (Figure S7c). This can be attributed to the superior LTP characteristics of the DS device compared with the shorter relaxation time of the ST device.

### Applications in Neuromodulation Function and Logic Gates

**Figure 6:**
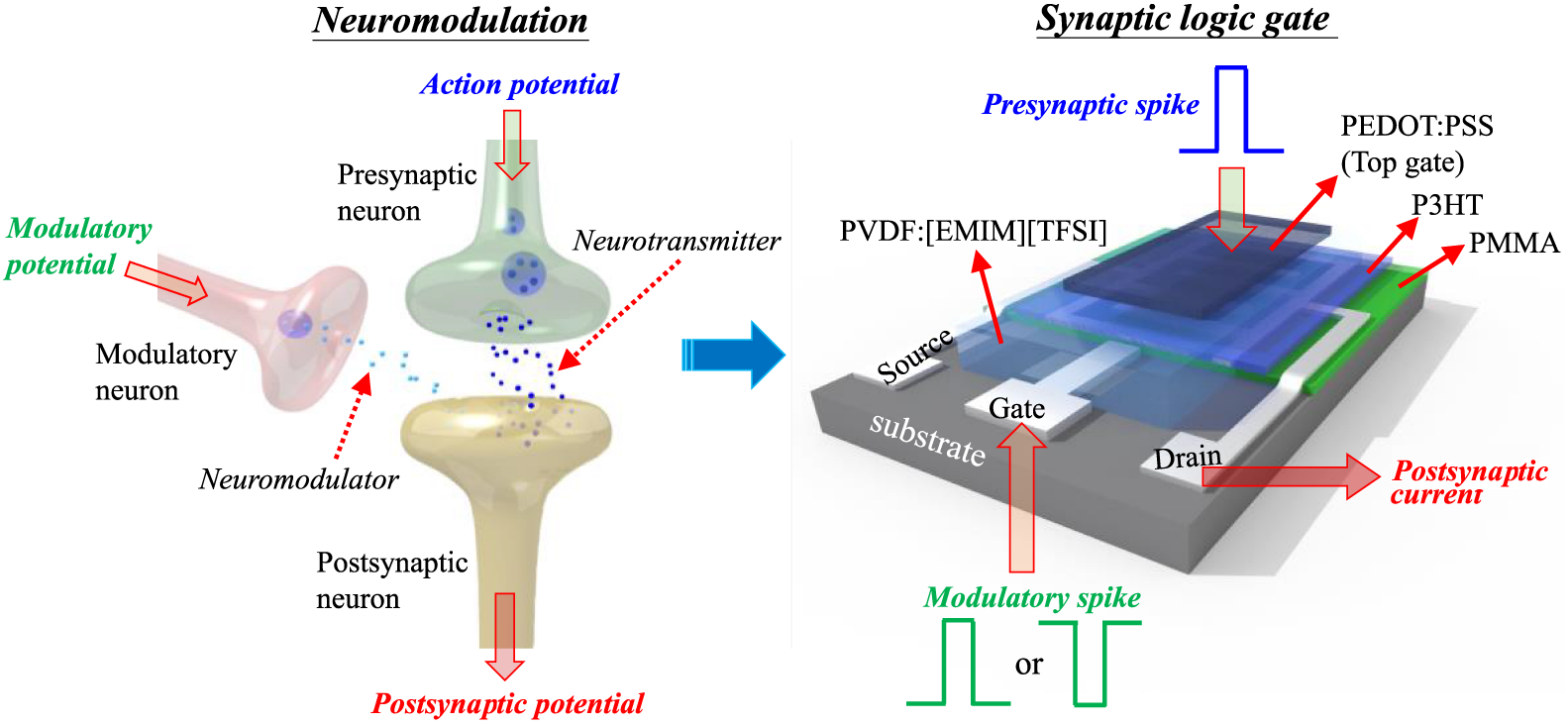
Illustration of the operation of a P3HT/PMMA PB-ESD synaptic dual-gate transistor analogous to neuromodulation in a biological synaptic neuron.

**Figure 7:**
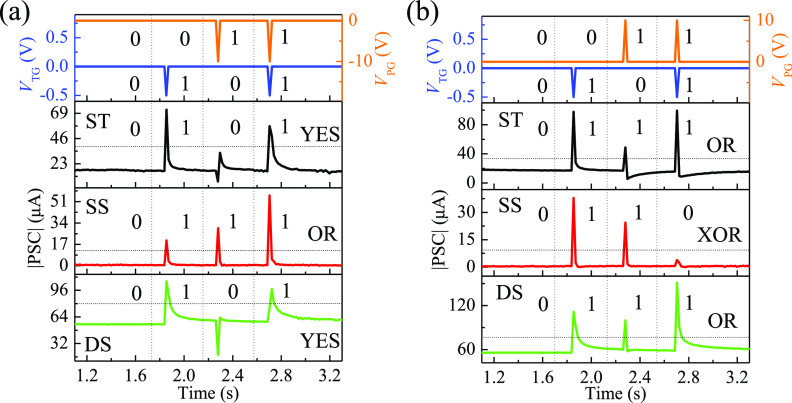
Logical operations of different P3HT/PMMA PB-ESD synaptic dual-gate transistors under stimulation by *V*_PG_ and *V*_TG_ with (a) the same polarity and (b) opposite polarities. The drain voltage was maintained at −0.5 V.

In biological organisms, a physiological process named neuromodulation commonly exists in nervous systems and occurs in synaptic neurons, as illustrated in Figure 6. With additional modulatory neurons, synaptic neurons can implement neuromodulation to control cognition, endocrine, satiety, muscle and motor systems, body temperature, mood, and sleep, among other biological organisms, through the joint effects of neurotransmitters and neuromodulators on the postsynaptic neuron. Combining two effects to trigger the next actions is like the operation of two-input logic gates in digital circuits. Hence, with an additional planar gate electrode to modulate the conducting channel, like the function of a modulatory neuron, these P3HT/PMMA PB-ESD synaptic transistors can emulate functions of neuromodulation to work as two-input synaptic logic gates (Figure 6). Figure 7 shows PSC variations of different P3HT-based synaptic transistors stimulated by various combinations of planar gate and top gate voltages (*V*_PG_ and *V*_TG_). For the condition of both negative *V*_PG_ and *V*_TG_ (Figure 7a), under the stimulation of only *V*_TG_ (inputs 0 and 1), all kinds of devices perform EPSC behavior (output 1). Under the stimulation of only *V*_PG_ (inputs 1 and 0), the ST and DS devices produce a PSC below the initial state, reflecting an inhibitory PSC behavior (output 0). In the device structure, the top gate and planar gate are located at the top and the bottom of the ion–gel dielectric, respectively (Figure 6). Same polarities of *V*_PG_ and *V*_TG_ cause opposite electric fields in the ion–gel dielectric. Therefore, negative *V*_PG_ depletes holes in the conducting channel, resulting in IPSC behavior of the ST and DS devices. As for the SS device, a negative *V*_PG_ can help release holes captured by PMMA into the conducting channel, leading to the observation of EPSC behavior (output 1). Under the stimulation of *V*_PG_ and *V*_TG_ (inputs 1 and 1), EPSC behavior (output 1) is observed in all kinds of devices. However, the opposite effects of *V*_PG_ and *V*_TG_ (hole depletion and hole accumulation) pose lower PSC values of the ST and the DS devices, compared with those stimulated by only *V*_TG_. The synergistic effect of *V*_PG_ and *V*_TG_ (increased holes in the conducting channel) causes higher PSC of the SS device, compared with that stimulated by only *V*_TG_. Based on the results of logical operations, the ST and DS devices can act as YES logic gates, and the SS device can operate as an OR logic gate. For the condition of positive *V*_PG_ and negative *V*_TG_ (Figure 7b), the stimulation of only *V*_TG_ (inputs 0 and 1) results in EPSC behavior (output 1) of all kinds of devices. The stimulation of only *V*_PG_ (inputs 1 and 0) also leads to the EPSC behavior (output 1) of all kinds of devices. Opposite polarities of *V*_PG_ and *V*_TG_ generate electric fields with the same direction in the ion–gel dielectric. Hence, the effects of positive *V*_PG_ and negative *V*_TG_ on the devices are identical. Nonetheless, the electric field of *V*_PG_ applied on the conducting channel is smaller than that of *V*_TG_, making PSC values of devices stimulated by *V*_PG_ lower than those stimulated by *V*_TG_. Under the stimulation of *V*_PG_ and *V*_TG_ (inputs 1 and 1), both ST and DS devices perform EPSC behavior (output 1) and their PSC values are higher than those under the stimulation of either *V*_TG_ or *V*_PG_ because of the synergistic effect of *V*_PG_ and *V*_TG_. Interestingly, no apparent increase is observed in the PSC of the SS device (output 0). The stimulation of *V*_PG_ or *V*_TG_ could cause holes to be captured by PMMA molecules nearby the conducting channel. The simultaneous stimulation of *V*_PG_ and *V*_TG_ could make PMMA capture enough holes to block hole accumulation, like the phenomenon observed in the SS device stimulated by 8 spikes (Figure 4d), and pose nearly unchanged PSC of the SS device. Based on the results of logical operations, the ST and DS devices can work as OR logic gates, and the SS device can perform XOR logical operation.

## Conclusions

Organic electrolyte-gated synaptic transistors based on P3HT/PMMA PB-ESD architecture with various microstructures, including ST, SS, and DS devices, were fabricated to study the neuromorphic behavior of devices. Among three kinds of devices, the ST device has poor quality of crystalline P3HT and flat interface of P3HT with ion–gel dielectric. The SS device has some PMMA domains in the P3HT layer and located near the P3HT/ion–gel dielectric interface. The DS device possesses larger amorphous P3HT portion, better quality of crystalline P3HT, and rough interface of P3HT with ion–gel dielectric. Under different conditions of stimulation, the ST device shows neuromorphic behavior of potentiated EPSC, PPF, and STP. Compared with the ST device, the DS device performs potentiated EPSC and PPF behavior, but the enhancement of PSC is higher, resulting from efficient charge transport and strong EDL due to better quality of crystalline P3HT and rougher P3HT/ion–gel interface, respectively. However, the DS device shows an LTP behavior because of prolonged hole release time induced by rough P3HT/ion–gel interface and large amorphous P3HT components. Unlike other two kinds of devices, the SS device shows neuromorphic behavior of depressed EPSC and PPD, stemming from suppressed hole accumulation by PMMA molecules near the P3HT/ion–gel interface. Moreover, like the ST device, the SS device performs an STP behavior as well. When applied to the simulation of neural networks with ANNs on the MNIST data set, both the ST and DS devices demonstrate high recognition accuracy (>83%). Specifically, the ST device achieves a recognition accuracy close to 90% when the *t*_off_ is short, while the DS device exhibits a better recognition accuracy when the *t*_off_ is longer. Additionally, with an additional planar gate electrode, these P3HT/PMMA PB-ESD synaptic dual-gate transistors can act like neuromodulation in biological synapses and work as two-input synaptic logic gates. Under the stimulation conditions of *V*_PG_ and *V*_TG_ with the same polarities, both ST and DS devices operate as YES logic gates, and the SS device works as an OR logic gate. Stimulated by *V*_PG_ and *V*_TG_ with opposite polarities, both ST and DS devices perform an OR logical operation, and the SS device demonstrates an XOR logical operation. A variety of neuromorphic behaviors of devices can be achieved under the same stimulation conditions by manipulating microstructural features of organic synaptic transistors. Microstructure and/or interface engineering can facilitate the development of organic synaptic transistors with electrical characteristics more analogous to biological neurobehaviors, which not only can promote the advancement of electronic devices with simultaneous features of computing, memory, and low power consumption, but can help develop biocompatible bionic prosthetics, advanced healthcare devices, and bionic artificial neural networks.

## Supporting Information Available

Supplemental electrical characterization of the as-prepared organic synaptic transistors, a comparative table of the extracted electrical parameters, the *G* ratio of the SS and ST devices relative to the DS device; supplementary absorption spectra, comparison of electrical and synaptic characteristics of the most related references and the current study, the EPSC curves obtained from single-spike and multiple-spike stimulations for the selected time duration and interval, demonstration of long-term potentiation (LTP) characteristics of the DS device under the application of 800 consecutive pulse stimuli, and results of the simulation of recognition accuracy under two stimulus conditions (PDF)
