# Supplementary material for: Modulating Neuromorphic Behavior of Organic Synaptic Electrolyte-Gated Transistors Through Microstructure Engineering and Potential Applications
Source: ACS Appl Mater Interfaces. 2024 Jul 26;16(31):41211–22. doi: 10.1021/acsami.4c05966 (PMC11310909; doi:10.1021/acsami.4c05966)
Supplement: Supplementary file 1 — am4c05966_si_001.pdf [file am4c05966_si_001.pdf]

Supporting Information

Modulating Neuromorphic Behavior of Organic Synaptic Electrolyte-Gated Transistors Through Microstructure Engineering and Potential Applications

Fu-Chiao Wu, [†] *Chun-Yu Chen,* [†] *Yu-Wu Wang,* [§] *Chun-Bin You,* [†] *Li-Yun Wang,* [†] *Jrjeng Ruan,* [‡] *Wei-Yang Chou,* [†] *Wei-Chih Lai,* [†] *and Horng-Long Cheng* [†,*]

[†] Department of Photonics, Meta-nanoPhotonics Center, National Cheng Kung University, Tainan 701, Taiwan

[‡] Department of Materials Science and Engineering, National Cheng Kung University, Tainan 701, Taiwan

[§] Institute of Photonics, National Changhua University of Education, Changhua 500, Taiwan

Corresponding Author

*Horng-Long Cheng, Email: shlcheng@ncku.edu.tw

Outline

1. **Supplementary electrical characterization of organic synaptic transistors**
2. **Supplementary absorption spectra**
3. **Comparison of electrical and synaptic characteristics of the most related references and the current study**
4. **Supplementary artificial synaptic characteristics of organic synaptic transistors**
5. **Application in the simulation of neural networks**

1. Supplementary electrical characterization of organic synaptic transistors

Table S1. Comparison of threshold voltage (V_t), subthreshold swing (S), on/off ratio, and maximum channel conductance (G_{max}) for various P3HT/PMMA PB-ESD-based organic synaptic transistors operated at $V_D = -0.5$ V.

Device	V_t (V)	S (V/dec)	On/off ratio	G_{max} (S/m)
ST	0.107 ± 0.048	0.168 ± 0.049	~ 5 × 10^2	5.09
SS	0.043 ± 0.026	0.173 ± 0.039	~ 2 × 10^2	0.05
DS	-0.133 ± 0.044	0.095 ± 0.021	> 5 × 10^4	> 100

Ps. At least 5 batches of devices were used to observe the standard deviation.

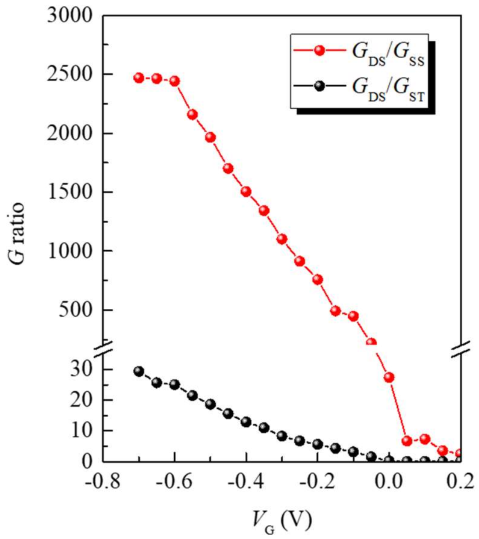

Figure S1. Comparison of channel conductance (G) value as a function of gate voltage (V_G). The G ratio is calculated as the G value of the DS device divided by the G values of the SS and ST devices, respectively, represented as G_{DS}/G_{SS} and G_{DS}/G_{ST}.

2. Supplementary absorption spectra

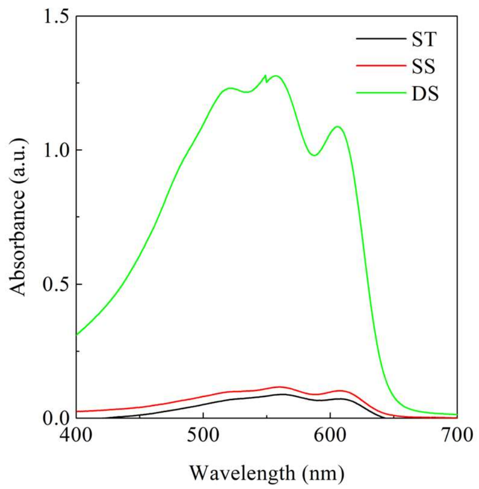

Figure S2. Absorption spectra of different P3HT specimens.

3. Comparison of electrical and synaptic characteristics of the most related references and the current study

Table S2. Comparison of electrical parameters [threshold voltage (V_t), subthreshold swing (S), on/off ratio, maximum channel conductance (G_{max}), and field-effect mobility] between previous reports on P3HT-based electrolyte-gated transistors and the current study.

Method, solvent, concentration, *W/L* [a]	V_{th} (V)	S (mV/dec)	On/off ratio	G_{max} (S/m)	Mobility (cm^2/Vs)	Ref.
Spin-coating, chlorobenzene, 0.91 wt%, 1000 μm/50 μm	–0.94	–	> 10^3	13.3	5.04 at V_D = –1.5 V	(s1)
Spin-coating, chlorobenzene, 0.91 wt%, 1 mm/50 μm	–1.18	–	> 10^4 – 10^4	50	3.97 at V_D = –1.0 V	(s2)
Spin-coating, chlorobenzene, 0.91 wt%, 3 mm/70–90 μm	> –0.6	100–150	> 6 × 10^3	15.6	20.24 at V_D = –0.4 V	(s3)
Spin-coating, chloroform, 0.2 wt%, 1000 μm/100 μm	–	–	> 10^5	20	2.83 at V_D = –0.1 V	(s4)
Spin-coating, chlorobenzene, 0.91–0.09 wt%, 1 mm/10 μm	–0.55	200	~ 10^6	80	2.25 at V_D = –1.5 V	(s5)
Spin-coating, chloroform, 0.68 wt%, 1 mm/100 μm	–0.13	–	~ 5 × 10^3	~10	1.13 at V_D = –1.0 V	(s6)
Spin-coating & Transfer-stamping, chloroform, 0.2 wt%, 1000 μm/100 μm	0.19	–	> 10^4	16	2.81 at V_D = –0.5 V	(s7)
Electrohydrodynamic jet printing, toluene, 0.2 wt%, 150 μm/300 μm	–0.83	73 ± 11	~ 10^5	22	0.12 at V_D = –0.1 V	(s8)
Drop-casting, p-xylene, 0.1 wt%, 2000 μm/200 μm	–0.13	95 ± 2.1	> 5 × 10^4	> 100	> 10 at V_D = –0.5 V	This work

[a] *W/L*: channel width and channel length of transistors.

Table S3. Comparison between previous reports on polymer-based electrolyte-gated transistors and the current study.

Seimconductors	Method, concentration	Minmum spkie voltage with width	V_{DS} (V)	PPF (%)	Long-term potentition and memory [a]	Maximun pulse number of multiple spike	Ref.
PDPP-DTT [b]	Spin-coating, 0.27 wt%	-3 V, 200 ms	-0.2	194	LTP	500	(s9)
DPP-based polymers [b]	Spin-coating and transfer 0.42 wt%	-0.7 V, 100 ms	-0.5	265	LTP	70	(s10)
P3HT	Spin-coating, 1.15 wt%	-1.0 V, 100 ms	-0.5	195	LTM	100	(s11)
P3HT with azide crosslinker	Spin-coating, 0.2 -0.87 wt%	-3.0 V, 50 ms	-0.01	200	LTP	100	(s12)
DPP-DTT [b]	Spin-coating, 0.62 wt%	-3.0 V, 130 ms	-0.1	~ 95	LTP	75	(s13)
P3HT with 2Bx crosslinker	Spin-coating, 0.34 wt%	-3.0 V, 30 ms	-0.01	-	LTM	50	(s14)
PDPP-TTVTT [b]	Spin-coating, 0.34 wt%	-2.0 V, 100 ms	-0.1	160 – 180	LTM	50	(s15)
P3HT	Drop-casting, 0.1 wt%	-0.5 V, 5 ms	-0.5	220	LTM	800	This work

[a] Long-term memory (LTM) is defined as the ability of a device to maintain the change in its conductance for an extended period after the stimulation is stopped, without spontaneous decay or reverting back to its initial state, or the ability to sustain the change for a relatively long duration, ranging from a few hours to several days or even longer.

[b] Abbreviations of polymer materials**:** PDPP-DTT: poly[2,5-(2-octyldodecyl)-3,6-diketopyrrolopyrrole-alt-5,5-(2,5-di(thien-2-yl)thieno [3,2-b]thiophene)]; DPP: diketopyrrolopyrrole; DPP-DTT: poly[2,5-(2-octyldodecyl)-3,6-diketopyrrolopyrrole-*alt*-5,5-(2,5-di(thien-2-yl)thieno[3,2-bthiophene)]; PDPP-TTVTT-β-$C_{10}C_{12}$: poly[2,5-bis(2-decyltetradecyl)pyrrolo[3,4-c]pyrrole-1,4-(2H,5H)-dione-(E)−1,2-di(2,2’-bithiophen-5-yl)ethene]; PDPP-TTVTT-C_{12}-TEG: poly[2,5-bis(2,5,8,11-tetraoxatricosan-23-yl)pyrrolo[3,4-c]pyrrole-1,4-(2H,5H)-dione-(E)−1,2-di(2,2’-bithiophen-5-yl)ethene]).

4. Supplementary artificial synaptic characteristics of organic synaptic transistors

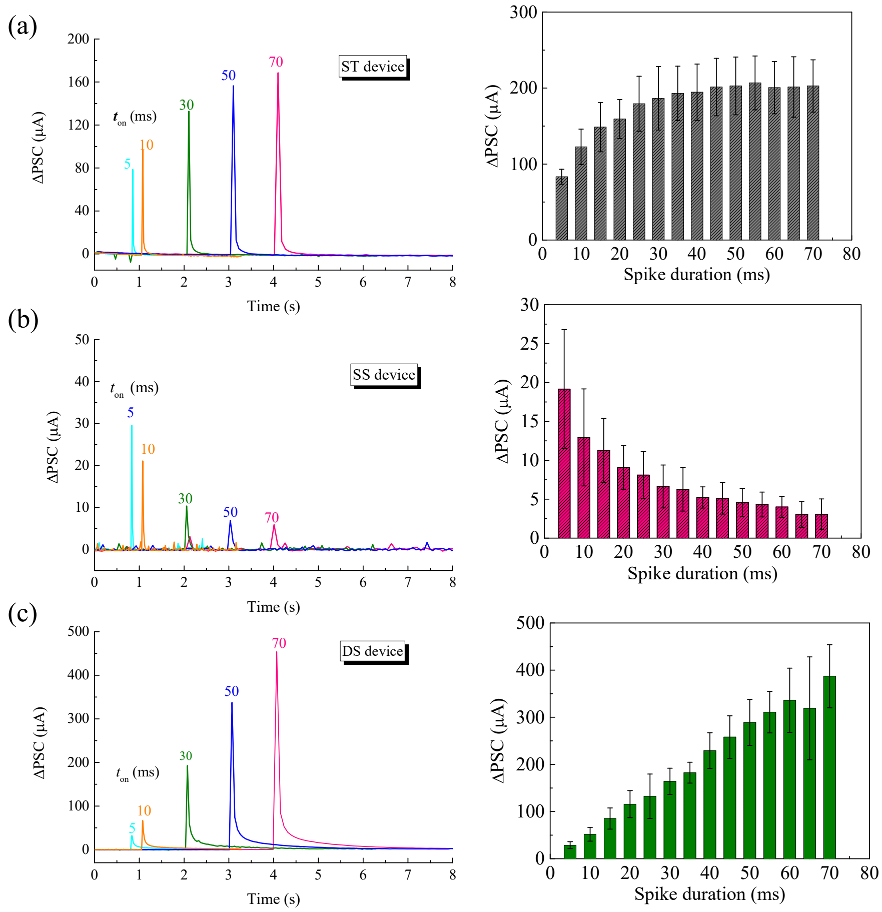

Figure S3. EPSC characteristics of (a) ST, (b) SS, and (c) DS devices stimulated by a single spike (V_G = −0.5 V) and using a source-drain voltage (V_D) of −0.5 V for reading. Left panels: EPSC responses for the selected spike durations (t_{on}). Right panels: Device-to-device variations of EPSC responses under the application of different spike durations.

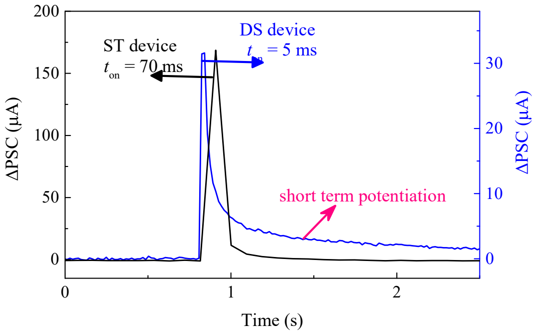

Figure S4. Comparison of EPSC curves of the ST and DS devices under a single spike (V_G = −0.5 V) for the selected on-time (t_{on}). The EPSC curve of the DS device exhibits significant short-term potentiation.

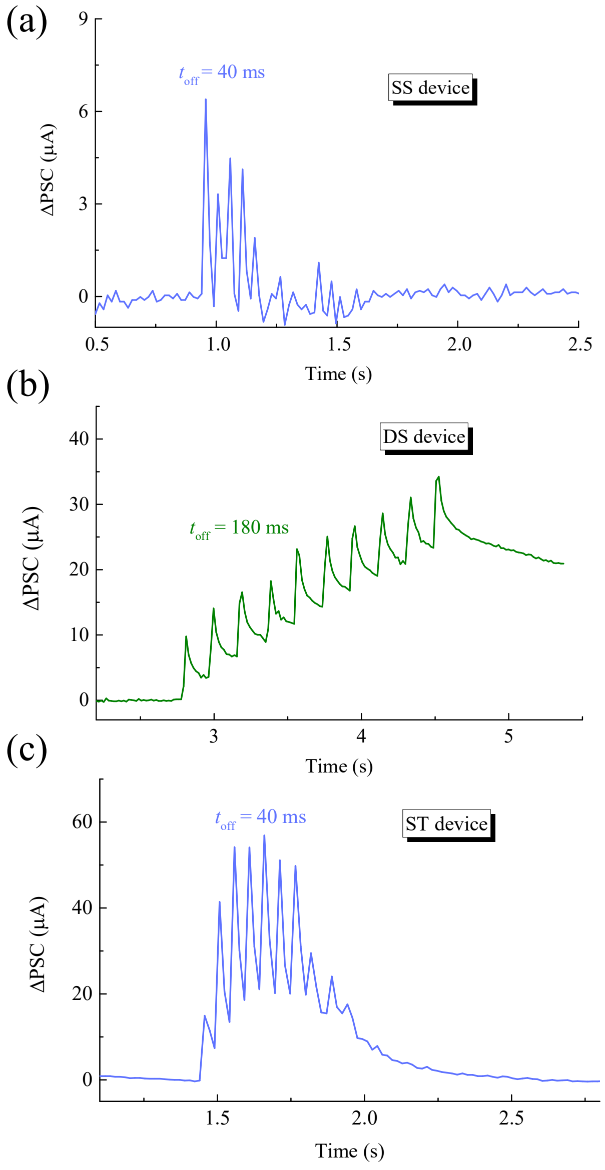

Figure S5. PSC variations of (a) SS, (b) DS, and (c) ST devices stimulated by 10 spikes ($V_G = -0.5$ V) with a time duration of 5 ms. The time interval between spikes is shown in the Figure. The PSC was read using a source-drain voltage (V_D) of −0.5 V.

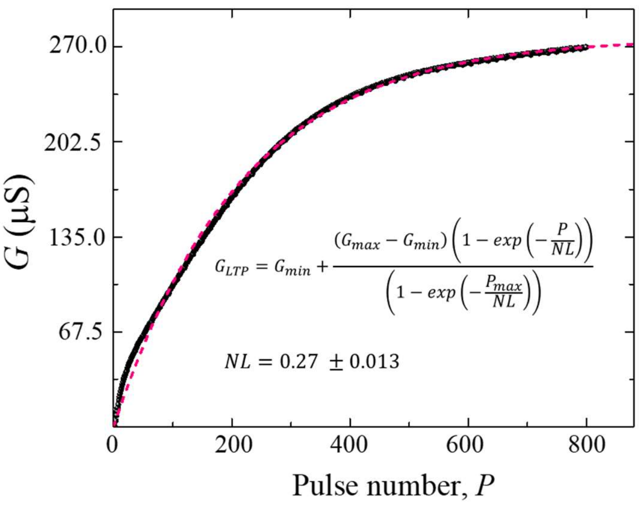

Figure S6. Demonstration of LTP characteristics of the DS device under the application of a large number of stimuli (V_G = −1.0 V), up to 800 times. The channel conductance (*G*) was read using a V_D of −0.5 V. The data can be well-fitted using the common model of the conductance change of LTP with the number of pulses (*P*). The extracted nonlinearity (NL) is also shown.

5. Application in the simulation of neural networks

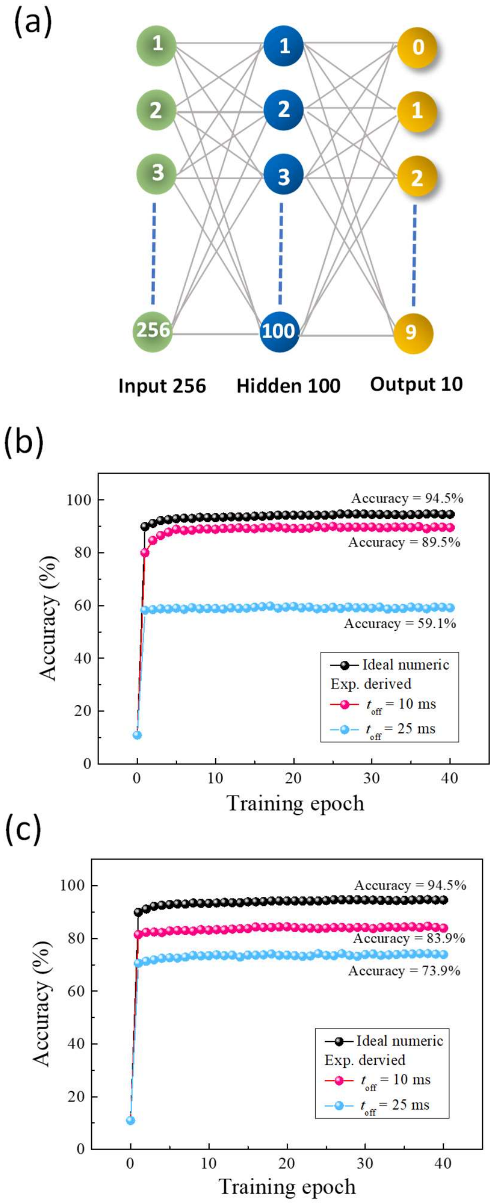

Figure S7. (a) Schematic of a three-layer artificial neural network with input, hidden, and output layers. Simulation of recognition accuracy as a function of training epoch for the (b) ST and (c) DS devices, considering the synaptic characteristics of a single device under two stimulus conditions: a spike duration of 5 ms at $V_G = -0.5$ V and different time intervals between spikes (t_{off}).

Reference

(s1) Nketia-Yawson, B.; Ahn, H.; Jo, J. W. Understanding Effects of Ion Diffusion on Charge Carrier Mobility of Electrolyte-Gated Organic Transistor Using Ionic Liquid-Embedded Poly(3-hexylthiophene). *Adv. Funct. Mater*. **2022**, *32*, 2108215,

(s2) Nketia-Yawson, B.; Lee, J. H.; Tabi, G. D.; Opoku, H.; Lee, J. J.; Ahn, H.; Jo, J. W. Stable Electrolyte Dielectric Engineered Bottom-Gate Poly(3-Hexylthiophene) Transistors with Enhanced Mobility, *Org. Electron.* **2022**, *102.* 106430.

(s3) Yadav, Y.; Ghosh, S. K.; Singh, P. High-Performance Organic Field-Effect Transistors Gated by Imidazolium-Based Ionic Liquids. *ACS Appl. Electron. Mater.* **2021**, *3*, 1496−1504.

(s4) Cho, K. G.; Cho, Y. K.; Kim, J. H.; Yoo, H.; Hong, K.; Lee, K. H. Thermostable Ion Gels for High-Temperature Operation of Electrolyte-Gated Transistors. *ACS Appl. Mater. Interfaces* **2020**, *12*, 15464−15471

(s5) Nketia-Yawson, B.; Tabi, G. D.; Noh, Y.-Y. Polymer Electrolyte Blend Gate Dielectrics for High-Performance Ultrathin Organic Transistors: Toward Favorable Polymer Blend Miscibility and Reliability. *ACS Appl. Mater. Interfaces* **2019**, *11*, 17610−17616.

(s6) Na, Y.; Kim, F. S. Nanodroplet-Embedded Semiconducting Polymer Layersfor Electrochemically Stableand High-Conductance Organic Electrolyte-Gated Transistors. *Chem. Mater*. 2019, 31, 4759−4768.

(s7) Cho, K. G.; Kim, H. J.; Yang, H. M.; Seol, K. H.; Lee, S. J.; Lee, K. H. Sub-2V, Transfer-Stamped Organic/Inorganic Complementary Inverters Based on Electrolyte-Gated Transistors. *ACS Appl. Mater. Interfaces* **2018**, *10*, 40672−40680.

(s8) Jung, E. M.; Lee, S. W.; Kim, S. H. Printed ion-gel transistor using electrohydrodynamic (EHD) jet printing process, *Org. Electron*. **2018**, *52*, 123–129.

(s9) Bhunia, R.; Kim, J. S.; Kweon, H.; Kim, D. J.; Kim, D. H. Ferroelectric Ion Gel-Modulated Long-Term Plasticity in Organic Synaptic Transistors. *Mater. Chem. Phys.* **2022**, *287*, 126227.

(s10) Chen, J.; Huang, W.; Zheng, D.; Xie, Z.; Zhuang, X.; Zhao, D.; Chen, Y.; Su, N.; Chen, H.; Pankow, R. M.; Gao, Z.; Yu, J.; Guo, X.; Cheng, Y.; Strzalka, J.; Yu, X.; Marks, T. J.; Facchetti, A. Highly Stretchable Organic Electrochemical Transistors with Strain-Resistant Performance. *Nat. Mater.* **2022**, *21*, 564−571.

(s11) Kim, D. W.; Yang, J. C.; Lee, S.; Park, S. Neuromorphic Processing of Pressure Signal Using Integrated Sensor-Synaptic Device Capable of Selective and Reversible Short- and Long-Term Plasticity Operation. *ACS Appl. Mater. Interfaces* **2020**, *12*, 23207–23216.

(s12) Choi, Y.; Oh, S.; Qian, C.; Park, J.-H.; Cho, J. Vertical Organic Synapse Expandable to 3D Crossbar Array. *Nat. Commun.* **2020**, *11*, 4595.

(s13) Wang, X.; Lu, W.; Wei, P.; Qin, Z.; Qiao, N.; Qin, X.; Zhang, M.; Zhu, Y.; Bu, L.; Lu, G. Artificial Tactile Recognition Enabled by Flexible Low-Voltage Organic Transistors and Low-Power Synaptic Electronics. *ACS Appl. Mater. Interfaces* **2022**, *14*, 48948−48959.

(s14) Choi, Y.; Ho, D. H.; Kim, S.; Choi, Y. J.; Roe, D. G.; Kwak, I. C.; Min, J.; Han, H.; Gao, W.; Cho, J. H. Physically Defined Long-Term and Short-Term Synapses for the Development of Reconfigurable Analog-Type Operators Capable of Performing Health Care Tasks. *Sci. Adv.* **2023**, *9*, eadg5946.

(s15) Sung, M.-J.; Seo, D.-G.; Kim, J.; Baek, H. E.; Go, G.-T.; Woo, S.-J.; Kim, K.-N.; Yang, H.; Kim, Y.-H.; Lee, T.-W. Overcoming the Trade-off Between Efficient Electrochemical Doping and High State Retention in Electrolyte-Gated Organic Synaptic Transistors. *Adv. Funct. Mater.* **2023**, 2312546.
